# PRIMER: Premedical Introduction to Mentored Emergency Research: Connecting Undergraduate Assistants and Emergency Medicine Residents in a Community Hospital Setting

**DOI:** 10.5070/M5.52386

**Published:** 2026-07-31

**Authors:** M Kaitlin Parks, Justin L Cupps, Jessica Meador, Melody Kashef, Danielle R Kirsch, Sonja Settle

**Affiliations:** * Norman Regional Hospital System, Department of Emergency Medicine, Norman, OK; ^ Oklahoma State University-Center for Health Sciences, Tulsa, OK; † University of Oklahoma College of Medicine, Oklahoma City, OK; ** University of Oklahoma, Norman, OK; †† Oklahoma State University Center for Health Sciences, Medical Library, Tulsa, OK; ^^ Oklahoma State University, Research Data Library Services, Stillwater, OK

## Abstract

**Audience and type of curriculum:**

This curriculum is designed to prime pre-medical-field undergraduate students with medical research concepts and skills necessary to assist Emergency Medicine (EM) residents on clinical research projects in a community hospital setting.

**Length of curriculum:**

The PRIMER lecture series should run two to three days with six hours of lecture content to be covered. Project completion and poster presentation should take 8–16 weeks depending on time constraints, resources, and project scope.

**Introduction:**

Scholarly research has taken an increasingly prominent role in both undergraduate and graduate medical education. However, researchers and clinician-teachers can be limited in productivity due to lack of funding, time, and formalized infrastructure. This can result in siloed activities, incomplete or unrealized ideas, and often make research challenging for student and resident physicians alike.^1^ These barriers to participating in scholarly research are further exacerbated for those in community-hospital-based residencies and community- or rural-medicine educational tracks because these facilities have further reduced access to research personnel and resources.^2^, ^3^ Simultaneously, research literacy and experience are becoming increasingly critical qualifications for admission to professional medical educational programs.^4^,^5^ Developing structured research training and mentorship for premedical students in community hospital settings can simultaneously enhance residents’ research capacity and provide premedical students with critical experience and exposure needed for medical school admission.

**Educational Goals:**

The purpose of this project is to design a didactic training to provide undergraduate research assistants the necessary training to enhance resident research projects in the emergency department.

**Educational Methods:**

The educational strategies used in this curriculum include: 1) Blended learning of in-person lectures and online readings and resources implemented in a three-day workshop to provide foundational research knowledge, skills, and resources to research assistants before beginning projects, 2) Experiential learning implemented in workshops interspersed through the three-day intensive, allowing students to begin literature searches, compose IRB proposals, and develop data management and analysis plans that apply to their assigned research project, and 3) Group Learning where students develop action plans, communication, and presentation skills.

**Research Methods:**

The educational outcomes were quantitatively and qualitatively analyzed by administration of a pre- and post-test and self-evaluation using a 5-item Likert scale as well as the completion of project and presentation of poster. Average examination scores and self-evaluation scores were analyzed for trends. Learner feedback was collected concurrently with the final assessment.

**Results:**

Average pre-test and self-evaluations scores were 3.29 ± 1.11 out of 7 and 11.0 ± 1.63 out of 20, respectively. Average post-test and self-evaluation scores increased dramatically to 5.71 ± 0.95 and 17.86 ± 1.77, demonstrating student acquisition of concepts and increased confidence in their knowledge of medical research. End-of-program scores were 6.43 ± 0.53 and 17 ± 1.15, respectively, and demonstrated good concept retention and retained confidence.

**Discussion:**

The PRIMER model serves to both increase the capacity for resident-driven research in community hospital settings and provide pre-professional students with training and experience to help matriculate and succeed in medical programs. Resident physicians (PGY1-PGY4, n=15) indicated that the PRIMER program would be very helpful in designing (n=12), implementing (n=12), and completing (n=13) a research project. We were successful in implementing the program with three of four projects completed in the initial time frame planned. One project was not completed, and one contributing factor was resident vacation time. Future iterations in our program will consider the full resident schedule for the duration of the program. Research assistants feedback suggested improvements on coordinating resident schedules (vacation and graduation) with the program. We plan to implement the program two to three times each year to accommodate the overwhelming undergraduate interest and further enhance our residents’ ability to ask clinically relevant research questions and explore those results in a community-based EM program.

**Topics:**

Medical research, statistics, data analysis, mentorship, data management, research ethics.

## USER GUIDE

**Table:** 

List of Resources:	
Abstract	1
User Guide	3
Didactics and Hands on Curriculum Chart	7
Appendix A: Suggested Three Day Intensive Schedule	11
Appendix B: Pre- and Post-test and Self-Evaluation	14
Appendix C: PRIMER: Principles of Clinical and Translation Research PowerPoint	16
Appendix D: PRIMER: Research Ethics PowerPoint	17
Appendix E: PRIMER: Literature Search and Citation Management PowerPoint	18
Appendix F: PRIMER: Basic Biostatistics Concepts PowerPoint	19
Appendix G: PRIMER: Data Collection and Management PowerPoint	20
Appendix H: PRIMER: Data Storage and Security PowerPoint	21
Appendix I: Suggested Deadlines for an Eight-Week Program	22
Appendix J: PRIMER Resources Estimate	23
Appendix K: PRIMER Learner Resources	25
K1: Pre-PRIMER CITI (Collaborative Institutional Training Initiative) Training	26
K2: Presentation Tips	27
K3: Abstract Writing Tips	29
K4: PRIMER Poster Template	31
K5: Student Worksheet PICOT Question Development	32
K6: PRIMER Recommended Reading and Reference List	34
Appendix L: PRIMER Facilitator Resources	37
L1: Study Question Development Workshop Facilitator’s Guide	38
L2: IRB Drafting Workshop Facilitator’s Guide	44

**Learner Audience:**

This curriculum is designed for undergraduate pre-medical-field, medical students, and residents.

**Length of Curriculum:**

PRIMER lectures and breakout groups should take approximately six hours of in-person time to execute. Project completion and poster presentation can range from 8 to 16 weeks depending on time constraints, resources, and project scope.

**Topics:**

Medical research, statistics, data analysis, mentorship, data management, research ethics.

**Objectives:**

By the end of the curriculum, students should be able to:

1. Identify common study designs in medical research and discuss commonly used statistical measures.
2. Discuss and apply the ethical principles governing medical research in human subjects.
3. Articulate and apply essential research data management skills, including file organization, data documentation, and data storage and security.
4. Effectively complete literature reviews and synthesize information from the current published body of scientific literature.
5. Compose elements of Institutional Review Board (IRB) proposal components.
6. Participate in the completion of a research project with emergency medicine residents and faculty.
7. Present a poster presentation of their project to residents and faculty.

### Brief introduction

Scholarly research has taken an increasingly prominent role in both undergraduate and graduate medical education. However, researchers and clinician-teachers are often siloed off from one another, and limited funding, time, and formalized infrastructure often make completing research challenging for student and resident physicians alike.^1^ These barriers to participating in scholarly research are further exacerbated for students in community-hospital-based residencies and community- or rural-medicine educational tracks because these facilities have further restricted access to research personnel and resources.^2^, ^3^ Simultaneously, research literacy and experience are becoming increasingly critical qualifications for admission to professional medical educational programs.^4^, ^5^ Developing structured research training and mentorship for pre-medical students in community hospital settings can simultaneously enhance residents’ research capacity and provide pre-medical students with critical experience and exposure needed for medical school admission.

### Problem identification, general and targeted needs assessment

While ample effort has been funneled into research rotation curriculum formation and revisions for emergency medicine residents,^6^–^10^ much of these curricula have been developed and implemented in academic medical centers. Community-hospital-based residencies often do not have the personnel or resources to develop or implement these types of rotations. The PRIMER curriculum offers a novel solution to that problem by partnering pre-medical-field with residents in community hospital settings, simultaneously increasing research capacity in these programs and developing effective future clinician-researchers. The curriculum provides pre-professional students with focused didactic education and the real-world skills necessary to matriculate into professional programs and participate in clinical research effectively during training. To the knowledge of the authors, no present curriculum like this has been published to date. PRIMER offers a first step into a new opportunity for community-hospital-based residencies to expand clinical research capacity in a novel way. The combined model of concise, didactic lectures, workshops, and experiential learning in a short period of time allows students to acquire, develop, and master the basics of design and analysis of clinical research. Essentially, this curriculum gives students the information necessary to partake in clinical research, allows them to immediately apply this information to the project they will present at the end of the curriculum, and then uses an experiential learning approach for them to develop and master the necessary skills to be good researchers. Program faculty become advisors and resources to students as they progress through the curriculum. This shifts responsibility, problem solving, and motivation to the student while helping them gain important clinical research skills. This affects students preparing to be clinicians as well as community hospitals positively, with the long-term outcome of producing research that can positively benefit patients in the community hospital setting.

### Goals of the curriculum

The purpose of this project is to design a didactic training to provide undergraduate research assistants with the necessary training to capably assist on resident research projects in the Emergency Department.

### Objectives of the curriculum

By the end of the curriculum, students should be able to:

1. Identify common study designs in medical research and discuss commonly used statistical measures.
2. Discuss and apply the ethical principles governing medical research in human subjects.
3. Articulate and apply essential research data management skills, including file organization, data documentation, and data storage and security.
4. Effectively complete literature reviews and synthesize information from the current published body of scientific literature.
5. Compose elements of Institutional Review Board (IRB) proposal components.
6. Participate in the completion of a research project with emergency medicine residents and faculty.
7. Present a poster presentation of their project to residents and faculty.

### Educational Strategies

See curriculum chart

This curriculum uses a combination of lectures, self-directed workshops, and guided experiential learning to impart and solidify necessary medical research concepts and apply skills and knowledge directly to research projects.

- *Lectures*
  The lecture series of this curriculum was developed *de novo* by program faculty and adjunct instructors to distill major topics in clinical research down to their necessary components to train able research assistants. Interactive lecture models were employed to ensure spaced repetition of concepts and increase learner engagement with important concepts. This approach ensured learners rapidly acquired content in study design, biostatistics, data management, and research ethics (Educational Objectives 1, 2 and 3).
- *Workshops*
  Intermittent workshops were used to break up lecture content and apply learned skills relating to literature search using clinical research databases, IRB composition and proposal, and designing research questions alongside residents. Research assistants were allowed to engage in a self-directed learning process; identifying gaps in their knowledge, developing problem-solving approaches, and conferring with their partnered resident and faculty to make progress on their project. This quickly formed and solidified skillsets related to Educational Objectives 4, 5 and 6.
- *Guided Experiential Learning*
  After the conclusion of the intensive, students were provided with a schedule of project deadlines for the duration of the curriculum, designated check-ins with faculty throughout the program, and given contact information of the program faculty. Students then began learning-by-experience as they applied concepts and skills from the intensive to their assigned research project. Faculty and resident mentors were made available to meet as needed to help troubleshoot issues, coordinate with Health Information Technicians (HIT) for data pulls, and answer questions that students had. This structure allowed the students freedom to problem-solve on their own while furnishing them with resources and assistance as they needed it. The final deadline was to submit a poster design detailing the research they had completed and present it to program faculty and residents (Educational Objective 6).

### Results and tips for successful implementation

The PRIMER curriculum was piloted with a group of eight research assistants and four residents over a period of nine weeks. The lecture series of the PRIMER curriculum was delivered over a three-day intensive of combined lecture and workshop time. Research assistants were given a pre- and post-test, as well as pre- and post-self-evaluations corresponding to educational outcomes before and after completing the intensive. Average pre-test and self-evaluations scores were 3.29 ± 1.11 out of 7 and 11.0 ± 1.63 out of 20, respectively. Average post-test and self-evaluations scores increased dramatically to 5.71 ± 0.95 and 17.86 ± 1.77, demonstrating student acquisition of concepts and increased confidence in their knowledge of medical research. End of program test scores and self-evaluations averages were 6.43 ± 0.53 and 17 ± 1.15, respectively, and demonstrate good concept-retention and retained confidence. The remaining course of the curriculum consisted of bi-weekly check-ins with research assistants to discuss progress, address challenges, and provide direction and support for students as needed. Research assistants were given the opportunity to give a practice presentation of their final poster in the penultimate week of the program. This gave the opportunity for them to receive peer and faculty feedback before presenting at the emergency medicine resident didactics.

We suggest the following strategies for successful implementation:

- Record audio and visual for the lectures throughout the intensive. Provide these recordings and the corresponding PowerPoint slides to research assistants to refer to as needed. Our program also benefitted from inviting research/data librarians and Institutional Review Board personnel from nearby university campuses to present topics within their areas of expertise. While we recognize that not all programs will have access to these kinds of personnel, we strongly recommend that programs seek out and take advantage of these potential partnerships where possible.
- Develop a presentation that addresses the steps to complete an IRB proposal specific to your institution as part of the three-day intensive (see Appendix A, Day 2). The original IRB Proposal presentation is not included in this publication because the specific details prevent the lecture from being generalizable to audiences beyond the institution.
- Allot ample time to develop proposed research projects with residents who will be paired with research assistants. Often Electronic Medical Record (EMR) access will be limited to preprofessional students, so residents may need to de-identify data before providing it to research assistants for analysis. This can be a common pitfall and create unnecessary slowdowns in project completion.
- Ensure that at least one program faculty member is available to meet with students throughout the self-directed learning portion of the curriculum, especially if the curriculum is being implemented over a shorter period.
- Allow students to select the project they wish to complete after the Principles of Clinical and Translational Research presentation, since this will enable them to connect content to experiential learning during workshops. Alternatively, students may assist their paired residents in developing the entire research question; however, we found that providing some initial ideas is effective given the limited medical knowledge of some.

### Evaluation and Feedback

In response to learner feedback, concepts in the Research Ethics and Principles of Clinical and Translational Research lectures were modified for clarity and relevance to the program objectives. Additionally, better guidance on converting qualitative data to quantitative data was incorporated into the Basic Biostatistics Concepts lecture. We will also consider students’ backgrounds in statistics or biostatistics when accepting students into future cohorts of the program. A question addressing student data management skills was added to the Self-Evaluation. Lastly, program faculty will be coordinating with resident schedules to ensure that the resident leading the project does not take extended vacation or graduate from the residency in the duration of the program to minimize logistical and communication issues for learners.

### Associated Content

Appendix A: Suggested Three-Day Intensive Schedule

Appendix B: Pre- and Post-test and Self-Evaluation

Appendix C: PRIMER: Principles of Clinical and Translation Research PowerPoint

Appendix D: PRIMER: Research Ethics PowerPoint

Appendix E: PRIMER: Literature Search and Citation Management PowerPoint

Appendix F: PRIMER: Basic Biostatistics Concepts PowerPoint

Appendix G: PRIMER: Data Collection and Management PowerPoint

Appendix H: PRIMER: Data Storage and Security PowerPoint

Appendix I: Suggested Deadlines for an Eight-Week Program

Appendix J: PRIMER Resources Estimate

Appendix K: PRIMER Learner Resources

K1: Pre-PRIMER CITI (Collaborative Institutional Training Initiative) Training
K2: Presentation Tips
K3: Abstract Writing Tips
K4: PRIMER Poster Template
K5: Student Worksheet PICOT Question Development
K6: PRIMER Recommended Reading and Reference List

Appendix L: PRIMER Learner Resources

L1: Study Question Development Workshop Facilitator’s Guide
L2: IRB Drafting Workshop Facilitator’s Guide

## Supplementary Information















## Figures and Tables

**Table:** 

Topic	Recommended Educational Strategy	Educational Content	Objectives	Learners	Timing, Resources Needed	Recommended Assessment, Milestones Addressed
Principles of Clinical and Translational Research	Interactive lecture with small-group case examples and discussion	Overview of case reports, observational and interventional study designs, phases of clinical trials, examples from literature	Differentiate between case reports, observational and interventional study designs. Identify appropriate research designs for various clinical questions. Describe the phases of clinical trials and their applications in translational research.	Pre- Medical Students and Medical Students	Time: 50 minutes (Two 25-minute lecture blocks) Equipment: PowerPoint, projector/screen	Assessment: Pre-, Post- and Final Quiz Milestones: SBP3 – Practice-based Learning and Improvement. Demonstrates the ability to appraise and apply medical literature. PBLI2 – Evidence-Based Practice: Integrates evidence into clinical decision-making.
Research Ethics, Bias, and Regulatory Frameworks	Case-based discussion and mini-lecture	Discussion of beneficence, justice, nonmaleficence and autonomy Definition of informed consent in the research process What is an IRB and why is it so important? Federally protected classes of people in clinical research Examples of ethical misconduct Discussing the need for protecting patient privacy	Explain the ethical principles (respect, beneficence, justice) governing human subjects research. Identify types of research misconduct and conflict of interest and describe strategies to mitigate them. Recognize vulnerable populations and apply IRB and informed consent principles appropriately.	Pre- Medical Students and Medical Students	Time: One 25- minute lecture block Instructors: 1–2 assistants for the workshop component are helpful Equipment: PowerPoint, projector/screen	Assessment: Pre-, Post- and Final Quiz Milestones: PROF1 – Professional Values: Maintains ethical principles in research and patient care. SBP2 – Systems-based Practice: Complies with institutional and regulatory requirements for research.
IRB Composition	Lecture detailing IRB components Hands-on workshop immediately after the lecture	Detail proper completion of each component of the institutional review board proposal *Note: Due to the highly variable nature of IRB proposal processes and forms, educators utilizing this curriculum will need to tailor this lecture and workshop to suit the IRB they will be functioning under.*	Explain the purpose and structure of the IRB and levels of review. Apply ethical principles and regulatory criteria to the development of an IRB protocol. Collaborate with peers to draft a real IRB submission for a student-led research project.	Pre- Medical Students Medical Students Residents assisting as mentors	Time: 30 minutes (lecture) Equipment: PowerPoint, projector/screen Time: 60 minutes (workshop) One to four faculty to learner ratio (Recommended resident for each project) Proper access to institutional review board proposal (internet access, appropriate login credentials, Microsoft Word document formats, etc.)	Assessment: Completion and submission of IRB draft form; faculty review of risk assessment and consent plan. Milestones: PROF1 – Professional Values: Demonstrates ethical principles in research conduct. SBP2 – Systems- Based Practice: Navigates institutional systems and regulatory requirements. PBLI2 – Evidence-Based Practice: Applies ethical and regulatory principles to research design.
Literature Search	Lecture demonstrating advanced search functions and resources for clinical research literature search. Hands-on workshop immediately after the lecture Guided experiential learning throughout PRIMER program	Demonstrate PubMed advanced search functions, including how to save searches Provide resources to compile relevant articles and citations (eg, PaperPile, Zotero) Provide library resources to source articles that are not publicly available	Conduct efficient literature searches using PubMed’s advanced features and filters. Apply search strategies to locate relevant literature for clinical and research questions.	Pre- Medical Students and Medical Students	Time: 30 to 45 minutes (lecture) Equipment: PowerPoint, projector/screen Time: 30 minutes (workshop) Equipment: Computers, internet access, shared software for citation management (if applicable)	Assessment: Pre-, Post- and Final Quiz Milestones: PBLI1 – Evidence Acquisition: Efficiently searches and appraises medical literature. ICS2 – Interprofessiona l and Team Communication: Communicates scholarly findings effectively.
Data Storage and Management	Lectures on data management and security practices in clinical research	Connect data privacy and security to PHI protection Describe organizational tools to manage multiple data files Provide ways to secure data physically and digitally throughout the research process	Articulate and apply essential research data management skills, including file organization, data documentation, and data storage and security.	Pre- Medical Students and Medical Students	Time: 60 minutes (two 30-minute lectures) Equipment: PowerPoint, projector/screen	Assessment: Learner self-evaluation on Pre-, Post-, and Final Quiz) Milestones: SBP2 – Systems-based Practice: Uses data responsibly and within regulatory frameworks. PBLI3 – Reflective Practice: Implements processes to improve research quality.
(Very) Basic Biostatistics	Lecture with interactive examples and data exercises	Descriptive vs inferential statistics, P-values, confidence intervals, t-tests, chi-square, ANOVA, regression	Summarize key descriptive and inferential statistics used in medical research. Interpret P-values and confidence intervals to assess study results. Identify common statistical tests (t-test, chi-square, ANOVA, regression) and their applications.	Pre- Medical Students and Medical Students	Time: 45 minutes) Equipment: PowerPoint, projector/screen	Assessment: Pre-, Post- Assessment and Quiz) Milestones: PBLI2 – Evidence-Based Practice: Applies statistical concepts to interpret research findings. SBP3 – Practice-based Learning and Improvement: Critically evaluates study validity and relevance.
Presentation of Clinical Research	End-of-curriculum presentation of research to program faculty and residents	Provide students an opportunity to synthesize and present their completed research in a professional manner Encourage students to answer questions pertaining to their research from faculty and residents	Present a poster presentation of their project to residents and faculty.	Pre- Medical Students and Medical Students	Time: 60 minutes (15 minutes per group) 10-minute presentation and 5 minutes for questions) (Recommended to be held during residency didactics for audience and participation) ) Equipment: PowerPoint, projector/screen	Assessment: Completion of poster and poster presentation session.

**Figure:**
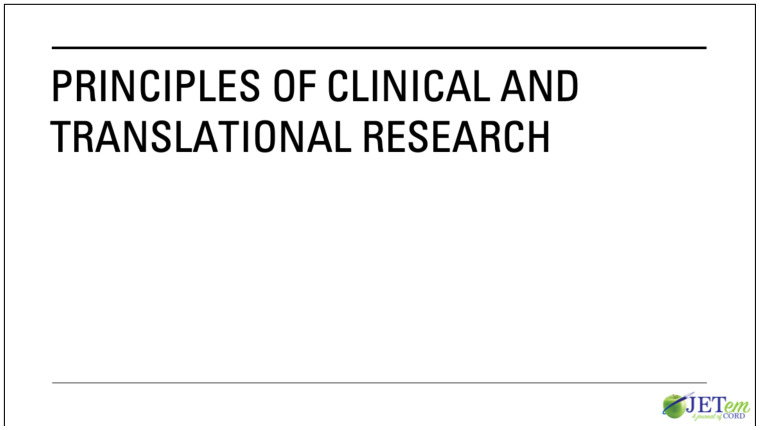
Please see associated PowerPoint

**Figure:**
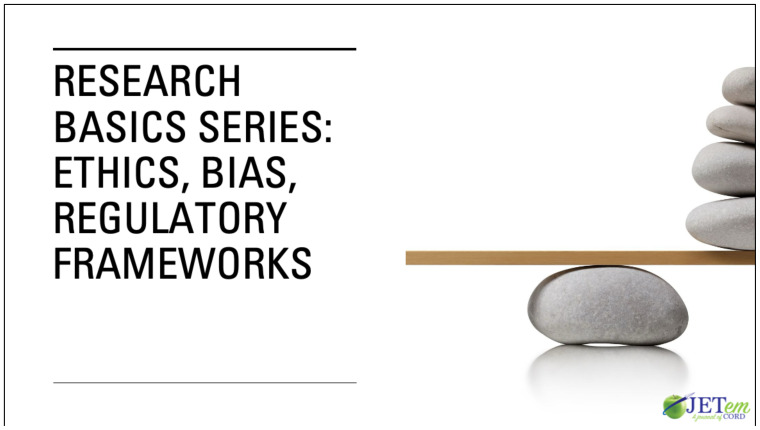
Please see associated PowerPoint

**Figure:**
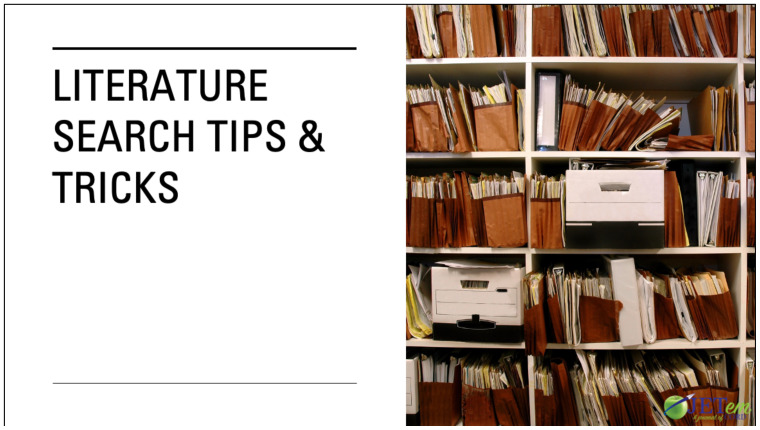
Please see associated PowerPoint

**Figure:**
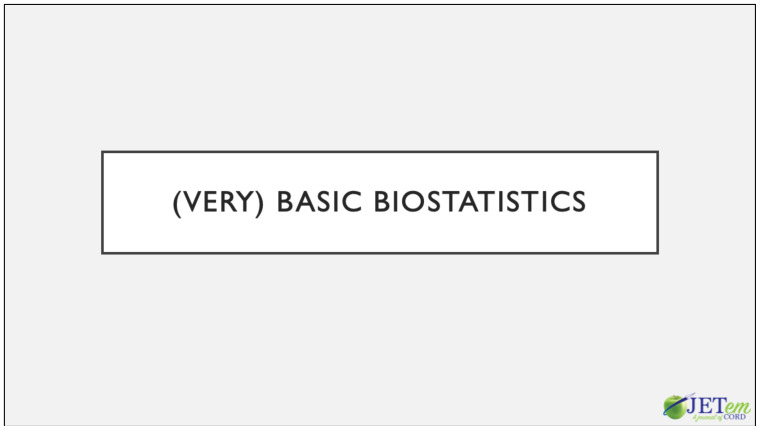
Please see associated PowerPoint

**Figure:**
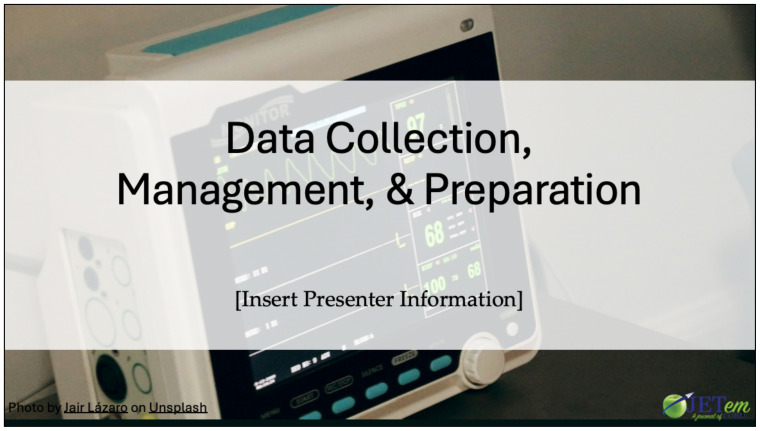
Please see associated PowerPoint

**Figure:**
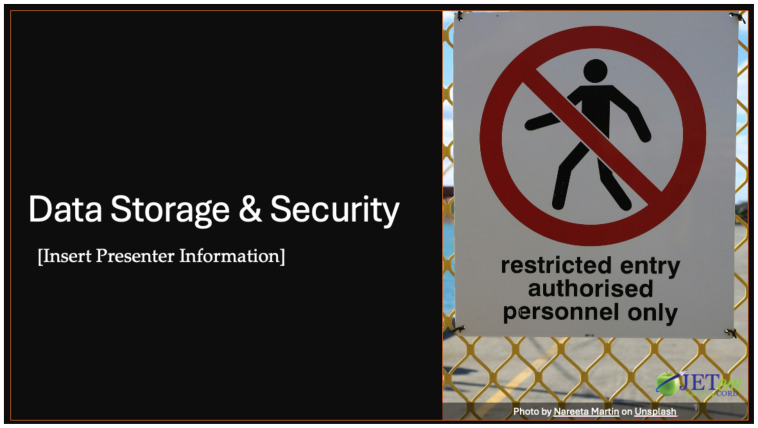
Please see associated PowerPoint

**Figure:**
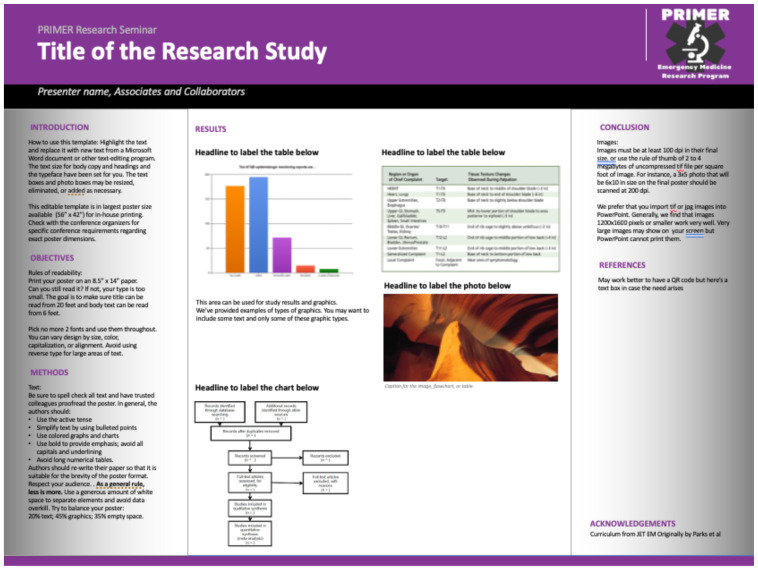
Please see associated PowerPoint

## Appendix A Suggested Three-Day Intensive Schedule

**Day 1: Foundations of Clinical Research (3 hours)**

**Objective**: Ground students in research basics, ethics, and project overview.

***Agenda:***

- **Welcome and Program Overview** (15 min)
  ○ Introductions, program goals, expectations
  ○ Final deliverable: Poster presentation
  ○ GroupMe
- **Principles of Clinical and Translational Research** (45 min)
  ○ Types of clinical research (observational, interventional)
  ○ Translational pipeline overview (T0–T4)
- **Research Ethics & Regulatory Frameworks** (30 min)
  ○ IRBs, informed consent, vulnerable populations
  ○ Recap of required CITI modules
  ○ Bias in observational studies
- **Study Design Workshop** (60 min)
  ○ Define research question using PICOT
  ○ Overview of study variables, hypotheses, outcomes
  ○ Exercise: Apply PICOT to each student’s assigned study
- **Q&A and Preview of Day 2** (15 min)
  ○ Individual groups exchange contact information

**Day 2: Practical Research Skills (2.5 hours)**

**Objective**: Train students in methodology, data planning, and literature tools.

***Agenda:***

- **Literature Review & Citation Management** (30 min)
  ○ How to conduct systematic searches (PubMed, Google Scholar)
- **Basic Biostatistics Concepts** (30 min)
  ○ Descriptive stats, p-values, confidence intervals
  ○ Statistical tests overview for typical clinical studies
- **IRB Components Deep Dive** (30 min)
  ○ Protocol template Overview
  ○ How to write the scientific background & significance
  ○ Consent forms, recruitment materials
- **IRB Breakout Session (Guided Drafting)** (60 min)
  ○ Each student group begins filling out IRB protocol for their assigned study
  ○ Faculty/mentor float between groups

**Day 3: Data Management, IRB, timelines**

**Objective**: Prepare IRB materials and timelines.

***Agenda:***

- **Data Collection and Management** (30 min)
  ○ Designing Collection process (chart reviews, case reports)
  ○ Excel: data entry best practices
- **Data Storage and Security** (30 min)
  ○ File Naming and Folder Organization
  ○ Human Specific Security Concerns
- **Timeline and Expectations Review** (10 min)
  ○ IRB submission timeline
  ○ Poster format, deadlines
  ○ Overview of bi-monthly check-in expectations
    ■ Schedule these with groups
- **General Work Session Continued** (60 min)
  ○ Space available for groups to collaborate
  ○ Faculty Assistance available

**Overview**

**Week 1: IRB drafted, submitted by early week 2**

**Week 2: IRB Submitted, Background Research complete**

**Week 3: Methods and data plan complete**

**Week 4–6: Data Collection**

**Week 7: Initial Results Summarized, Abstract completed, Poster outline drafted**

**Week 8: Final Poster Completed, poster presentation practice +/− showcase**

## Appendix B PRIMER Pre- and Post-test with Self-Evaluation

1. Which of the following is not a principle of ethical research in human subjects?
  - Beneficence
  - Justice
  - Respect for Persons
  - Objectivity
2. The alpha value describes:
  - The probability of making a Type II error
  - The threshold for significance
  - The probability of failing to reject the null hypothesis when it is false
  - None of the above
3. Phase 3 clinical trials compare novel treatments to:
  - The standard of care
  - No treatment
  - Another experimental treatment
  - All of the above
4. Which of the following includes blinding patients to their assigned treatment group?
  - Single-blind trials
  - Double-blind trials
  - Triple-blind trials
  - All of the above
5. Which of the following is not a protected group?
  - Pregnant women
  - Prisoners
  - Children
  - College students
6. Which of the following study designs is most helpful in characterizing rare diseases?
  - Case-control study
  - Cross-sectional study
  - Cohort study
  - Case report
7. Which statistical test compares variance between three or more groups?
  - T-test
  - ANOVA
  - Chi-squared
  - Chronbach’s alpha

Self-evaluation:

**Table:**

1. I can describe the ethical principles of research in human subjects.				
Strongly Disagree	Somewhat Disagree	Neutral	Somewhat Agree	Strongly Agree
2. I understand the design and application of research study models in medical research.				
Strongly Disagree	Somewhat Disagree	Neutral	Somewhat Agree	Strongly Agree
3. I can collect, manage, and store data in an organized manner for medical research purposes.				
Strongly Disagree	Somewhat Disagree	Neutral	Somewhat Agree	Strongly Agree
4. I can explain how common statistical measures are used in medical research and recognize their limitations.				
Strongly Disagree	Somewhat Disagree	Neutral	Somewhat Agree	Strongly Agree
5. I can identify the key components of an IRB proposal and accurately complete each section.				
Strongly Disagree	Somewhat Disagree	Neutral	Somewhat Agree	Strongly Agree

## Appendix I Suggested Deadlines for an Eight-Week Program

**Table:**

Week	Meeting Details	Deadline Date: Deadline/Milestone/Homework	Location
**Week #1**	RA Orientation #1 (9 am - 2 pm)		
**Week #1**		CITI Program Research Training Due by 5 PM	https://about.citiprogram.org/
**Week #1**	RA Orientation #2 (9 am - 2 pm)		
**Week #1**	RA Orientation #3 (9 am - 2 pm)		
**Week #2**	Check in with Research Leads	IRB drafted and submitted by early week 2	Zoom
**Week #2**		IRB Submitted/Background Research Complete	
**Week #3**	Check in with Research Leads	Research Summer Program Check-in 1	Zoom
**Week #3**		Methods and data plan complete	
**Week #4–6**		Data Collection	
**Week #7**		Initial Results Summarized, Abstract completed, Poster outline drafted	
**Week #8**		Final Poster Completed, poster presentation practice +/− showcase	Note: Date to be determined for the poster presentation

## Appendix J PRIMER Resources Estimate

For this cohort of 12, we had a faculty lead, faculty co-lead, two supporting faculty, and two external lecturers. Time to implement is an estimate since we were developing the program de novo.

**Table:**

Faculty	Time	Physical Resources	Other/Notes
Lead Faculty	25–30 hours over the course 3 hours in selection process and initial emails 16 hours in person Anticipated 2 hours lecture prep and 1 hour workshop facilitation prep Untracked time for email responses and occasional meetings with groups		This person was present at all sessions, gave two lectures, led the workshops and coordinated space reservation, offline assistance to teams, catering for sessions, poster printing, project ideas.
Co-Lead Faculty	10 hours over the entire course		This person was present at most in-person sessions, gave one lecture, and helped facilitate workshops, provided offline assistance to teams.
Adjunct faculty	0.5–1-hour lecture prep 1 hour in person for lecture 1–3 additional hours for some to help facilitate workshops		These attendings, residents, and one medical student with subject matter expertise were primarily engaged in a lecture topic and to help with one workshop session.
Lead Admin RA	20 hours		This assistant helped with answering emails and online questions, tracking CITI completion, and scheduling polls.
		Meeting space with projectors for the two in-person sessions and the final poster presentation	We chose to cater coffee for each in-person meeting and one meal (breakfast or lunch). This is optional and cost approximately $250 for breakfast/coffee and $400 for lunch.
		Posters printed	$35 each through private printer. Could be done digitally if needed.

## Appendix K PRIMER Learner Resources

K1: Pre-PRIMER CITI (Collaborative Institutional Training Initiative) Training

K2: Presentation Tips

K3: Abstract Writing Tips

K4: PRIMER Poster Template

K5: Student Worksheet PICOT Question Development

K6: PRIMER Recommended Reading and Reference List

### Appendix K1: Suggested Deadlines for an Eight-Week Program

If you have already completed, you can email a pdf to program leadership. If not:

1. Go to https://about.citiprogram.org/
2. Create an account.
3. Associate with your university by logging in with your student credentials
  - If you do not have access to a school program, reach out to program leadership.
4. Complete “Human Research - Group 3 Research Investigators and Key Personnel requiring both Biomedical and Social/Behavioral (ID 1503).”
5. Save a pdf of your completion certificate and email a copy to program leadership. Keep a copy for your own files. This will qualify you to participate in research at institutions across the country.

### Appendix K2: Presentation Tips

#### Poster Format

- You may use the provided poster template as your starting point.
- **Never use smaller than size 20 font** — bigger is better. Your audience needs to be able to read from a distance.
- Spell out abbreviations on first use. Spelling them out throughout looks more polished if you have the space.
- Use a QR code to link to detailed information (eg, full reference list) and save poster real space.
- If submitting to a specific conference, look up their formatting guidelines and apply them from the start.

#### Images

- Use high-resolution images only.
- **Avoid screenshots** — export your Excel results directly to a graph or image file for a much cleaner result.
- To find large images in Google: search your term → click **Tools** → **Size** → **Large**. Results will predominantly be high-resolution files that project well.
- **Cite your images.** Free and paid high-quality graphics are available from: Noun Project, Canva, Pixabay, and Adobe Stock.

#### PowerPoint Presentations

For specific medical conferences, always look up their submission guidelines. For the **PRIMER program conclusion presentation** at the residency conference or didactics, use the structure below.

#### Recommended Slide Structure

**Table:**

Slide	Content
**1**	Poster overview — start with your full poster so the audience orients to your work
**2**	Introduction & Objectives — introduce yourself, your team, and why you pursued this project
**3**	Methods
**4–5**	Results — consider two slides if your data is complex or visual
**6**	Discussion — include limitations and speak to next steps for the project
**7**	Conclusion
**8**	Poster overview again — end where you started

#### Delivery Tips

- There may be more text on your slides than in a typical talk — give your audience time (and silence) to read each slide before you move on.
- Include limitations when discussing your results.
- Speak to next steps and future directions for the project.
- Anticipate 2–3 audience questions and prepare answers with your team ahead of time.
- **Practice with your team** — consider assigning specific slides to different team members.

**Table:**

Preparing for Q&A
Think about what questions your faculty and residents are most likely to ask: ○ Why did you choose this study design? ○ What are the limitations of your data source? ○ What would you do differently if you repeated this study? ○ What are the clinical implications of your findings? Discuss these with your team before the presentation so everyone is ready to contribute.

### Appendix K3: Abstract Writing Guide

#### Word Count

Maximum word count is **250 words**. Some conference and journal submissions require as few as 150 words, though 250–350 is more common. Always check the specific guidelines for your target venue before you begin writing.

#### Abstract Sections

##### Title

A clear, descriptive title that reflects your study design, population, and main finding or question.

##### Authors

- **Author order** should reflect individual contributions and must be approved by PRIMER leadership.
- **First author:** Resident lead or lead research assistant.
  ○ **Second author:** Collaborating research assistant.
  ○ **Last author:** Faculty PI.
- Include professional titles for all authors (MD, DO, MPH, MS, BS, etc.).

##### Required Sections

**Table:**

Section	What to Include
**Introduction**	1–2 sentences of background information. End with your hypothesis and objectives clearly stated in the final sentence.
**Methods**	Study design. Where the study was conducted. How participants were selected (key inclusion/exclusion criteria). What data points were collected. How data were analyzed.
**Results**	Number of patients/encounters in your dataset. Key demographics (age, sex, etc.). Key findings.
**Conclusions**	Key findings. What you want others to take away from this work. Limitations of the study. Next steps or future directions.

#### Additional Resources

- Review these guidelines when preparing submissions for specific conferences:
- Council of Residency Directors guidelines for the Advances in Education Research and Innovation Forum. Recommended if submitting to a medical education venue.
- View CORD Research Abstract Guidelines
- Society for Academic Emergency Medicine scoring rubric for abstract submissions. Useful for understanding how reviewers evaluate your work.
- View SAEM Abstract Score Card (PDF)

Conferences you might want to consider submitting to concurrently or after the PRIMER presentation: AAEM, ACOEP, ACEP, CORD, SAEM, your own hospital or affiliated university (quality improvement or research symposium) and regional medical groups or associations (search city/county medical association, state medical association, regional trauma boards etc, and some projects may have more specialized relevance to EM subspecialities or more broad applicability to other medical organizations. See the resource for suggestions and a little about the organizations.

#### Appendix K6: PRIMER Recommended Readings and Reference List

Asynchronous Reading for Research Assistants

**Table:**

How to Use This List
These articles are assigned as asynchronous reading to complement the PRIMER intensive. You do not need to read every article in full before Day 1 — instead, use them as references throughout the program. Articles on writing and reporting are most useful when you begin drafting your manuscript or abstract.

**1. Study Design & Observational Research**

These readings support the Principles of Clinical and Translational Research lecture and the PICOT workshop. They will help you understand how to select an appropriate study design and identify the key features of the most common study types used in emergency medicine research.

1. Song JW, Chung KC. Observational studies: Cohort and case-control studies. *Plast Reconstr Surg*. 2010;126(6), 2234–2242.
  DOI: https://doi.org/10.1097/PRS.0b013e3181f44abc
   Excellent primer on the two most common observational study designs in clinical research. Covers study design diagrams, methodological issues, bias, and the STROBE reporting guidelines. Read before the PICOT workshop.
2. von Elm E, Altman DG, Egger M, Pocock SJ, Gotzsche PC, Vandenbroucke JP. Strengthening of the reporting of observational studies in epidemiology (STROBE) statement: Guidelines for reporting observational studies*. Ann Intern Med*. 2007;147(8):573–577.
  DOI: https://doi.org/10.7326/0003-4819-147-8-200710160-00010
   The 22-item STROBE checklist is the reporting standard for observational studies. Use this when writing your methods and results sections. Many journals now require STROBE compliance at submission.

**2. Survey Research**

These readings are relevant if your project involves a survey or questionnaire component. They cover the full survey research process from question design to analysis and reporting.

3. Jones TL, Baxter MA, Khanduja V. A quick guide to survey research*. Ann R Coll Surg Engl.* 2013; 95(1), 5–7.
  DOI: https://doi.org/10.1308/003588413X13511609956372
   A concise, practical overview of survey design, implementation, and analysis. Particularly useful for understanding validated instruments, question types, and strategies to improve response rates.
4. Kelley K, Clark B, Brown V, Sitzia J. Good practice in the conduct and reporting of survey research. *Int J Qual Health Care.* 2003;15(3), 261–266.
  DOI: https://doi.org/10.1093/intqhc/mzg031
   A step-by-step checklist for conducting high-quality survey research, with emphasis on avoiding common pitfalls in sampling, design, analysis, and reporting. Pairs well with the Jones et al quick guide above.

**3. Academic Writing & Abstract Submission**

These readings are assigned for the abstract writing and manuscript phase of the program. Read these after your data collection is complete and before drafting your abstract or poster.

5. Langdorf MI, Hayden SR. (). Turning Your abstract into a paper: Academic writing made simpler. *West J Emerg Med.* 2009;10(2), 120–123.
  https://westjem.com/article/turning-your-abstract-into-a-paper-academic-writing-made-simpler/
   A practical guide to academic writing written specifically for emergency medicine. Covers journal selection, brevity, structure, common writing errors, and how to respond to peer reviewer comments. Includes a manuscript template.
6. Society for Academic Emergency Medicine (SAEM). SAEM abstract scoring and submission guidelines. 2021, SAEM Annual Meeting. Available online:
  https://www.saem.org/meetings-and-events/annual-meeting/annual-meeting-navigation/education/abstracts
   The SAEM abstract scoring rubric evaluates clarity of objectives, methods, outcomes, data analysis, generalizability, relevance, innovation, writing quality, and strength of conclusions. Reviewing this before writing your abstract will help you understand how reviewers evaluate submissions.

**4. Additional Resources & Reporting Guidelines**

The following resources are recommended as reference tools rather than assigned reading. Bookmark them and return to them as needed during the project.

- **EQUATOR Network (equator-network.org):** The central hub for all reporting guidelines in health research. Includes STROBE, CONSORT (randomized trials), PRISMA (systematic reviews), SQUIRE (quality improvement), and more. Use this to confirm you are following the correct reporting standard for your study type.
- **STROBE Statement website (strobe-statement.org):** Provides separate checklists for cohort, case-control, and cross-sectional studies, plus the full Explanation and Elaboration article that walks through each checklist item with examples.
- **PubMed / NCBI (pubmed.ncbi.nlm.nih.gov):** Primary database for medical literature searches. Many PRIMER projects will rely on PubMed for background research and literature review. Refer to the Literature Search lecture and workshop for tips on using advanced search features and MeSH terms.
- **AMA Manual of Style (amamanualofstyle.com):** The standard citation and style guide for medical journals. Most EM journals follow AMA citation format. Free access may be available through your institution’s library.

## Appendix L Primer Facilitator Resources

L1: Study Question Development Workshop Facilitator’s Guide

L2: IRB Drafting Workshop Facilitator’s Guide

### Appendix L1: Study Question Development Workshop Facilitator’s Guide

**Table:**

**Duration**	45–60 minutes
**Timing**	Day 1 of PRIMER – Study Design Workshop (following the Principles of Clinical and Translational Research lecture)
**Faculty Needed**	Lead Faculty + 1–2 Support Faculty; EM resident mentors strongly encouraged to join
**Room Setup**	Small groups at tables
**Materials**	Printed PICOT worksheet (one per student, template provided in this guide) Project assignment summary sheet if developed (title, brief overview of each resident project) Laptops/tablets for students to begin background reading and drafting

**Learning Objectives**

By the end of this workshop, students will be able to:

- Identify and define each component of a PICO/PICOT framework (Population, Intervention/Issue, Comparison, Outcome, Time).
- Translate a broad clinical question into a structured, answerable PICOT question.
- Recognize which study design (observational vs. interventional) is appropriate for their proposed research question.
- Identify at least one data source or patient population relevant to their assigned project.
- Distinguish between feasible semester-length projects and projects requiring more resources or longer timelines.

**Background for Facilitators**

*What is PICOT?*

PICOT is a structured framework for asking clinical and research questions. It was originally developed to support evidence-based clinical practice and has been widely adopted in clinical research training. The acronym stands for:

**Table:**

Letter	Element	Definition & Prompts for Students
**P**	**Population**	Who is the study about? Define inclusion/exclusion criteria. Age group, diagnosis, care setting (eg, adult patients presenting to the ED with chest pain).
**I**	**Intervention / Issue**	What is the main exposure, treatment, test, or phenomenon being studied? For observational studies, this may be a risk factor or characteristic rather than a true “intervention.”
**C**	**Comparison**	What is the alternative? Standard of care, another treatment, no treatment, a different patient group, or historical controls. For descriptive studies, a comparison group may not apply.
**O**	**Outcome**	What are you measuring? Should be specific and measurable (eg, 30-day readmission rate, length of stay, mortality, need for dialysis etc).
**T**	**Time**	Over what period? How long will follow-up last? For retrospective chart reviews, this might be the date range of the data pull.

*Matching PICOT to Study Design*

After students formulate a PICOT question, help them verify that the study type fits. Use the examples below to frame the conversation:

**Table:**

Study Type	When to Use It	PRIMER Example
**Case Report / Series**	Novel presentation, rare diagnosis, unusual outcome. No comparison group needed.	A series of bupropion overdoses where whole bowel irrigation was used.
**Cross-Sectional**	Snapshot in time; prevalence of a condition or exposure. Good for administrative data.	What proportion of ED patients with sepsis received antibiotics within 1 hour in 2023?
**Retrospective Cohort**	Existing data; follow group forward from exposure to outcome. Feasible in one semester.	Do ED patients with diabetes have longer lengths of stay (LOS) than those without when presenting with infection?
**Case-Control**	Rare outcome; work backward from outcome to exposure.	Are patients who had adverse drug events more likely to have had incomplete allergy documentation?
**Prospective /Interventional**	Requires prospective enrollment and often full IRB approval. More difficult to do in a single semester but could extend past this formal cohort session	Does lidocaine, epinephrine, and tetracaine (LET) application in triage reduce LOS for uncomplicated lacerations? (Likely multisemester or

**Table:**

Facilitator Tip: Readily Available Data Sources
**Encourage students to consider the following data sources – these are often accessible with standard retrospective/exempt IRB review and can realistically be completed in one semester:** Using readily available data is not a requirement of the curriculum but offering suggested projects that would be more easily attainable can help students understand the scope of feasibility and ensure their success. **Electronic Medical Records (EMR) / Chart Review:** Retrospective pulls of de-identified patient data via HIT coordinators. Common for ED quality improvement, LOS, triage studies. **Quality Improvement / Dashboard Data:** Many hospitals generate monthly reports on metrics like door-to-ECG times, sepsis bundle compliance, fall rates. Requesting existing reports may be efficient. **Public Databases:** CDC WONDER, HCUP NIS (National Inpatient Sample), NAMCS (National Ambulatory Medical Care Survey). Publicly available, de-identified, free to access. Great for national-level prevalence or trend studies. **Published Clinical Datasets / Registries:** MIMIC-IV (critical care), PhysioNet, ACS NSQIP. Requires free credentialing but provides rich clinical data. **Survey / Chart Audit Studies:** Anonymous surveys of ED staff or simple chart audits of documentation compliance. These are often exempt or expedited IRB categories.

Remind students: EMR data pulls require coordination with the HIT team and the resident mentor. Build this into the Week 1–2 timeline and encourage early contact and check-ins.

**Session Plan**

*Segment 1: Introduction & Framing (10 minutes)*

Facilitator Script / Talk Track

“You’ve learned about the major types of clinical research – case reports, observational studies, and interventional trials. Now we’re going to take the question that’s at the heart of your project and turn it into something precise enough to study.”

Key Points to Cover

- Display the PICOT acronym with a plain-language definition of each element (slide or whiteboard).
- Acknowledge that not every element applies to every study – descriptive studies may lack a “C,” and T is optional but useful.
- You can use the following example to demonstrate or just initiate their discussions as a group to start the workshop with their forms as a starting point.

Worked Example to Project (display on screen)

**Broad Question:** Does using a sepsis protocol in the ED improve outcomes? **P:** Adult patients (≥18) presenting to the ED with suspected sepsis? **I:** Sepsis protocol bundle (early antibiotics, IV fluids, blood cultures within 1 hour) **C:** Standard care without formal protocol **O:** In-hospital mortality and 30-day readmission rate **T:** Retrospective review over 24 months (Jan 2022 – Dec 2023) **PICOT Question:** In adult ED patients with suspected sepsis, does adherence to a sepsis bundle protocol compared to standard care reduce in-hospital mortality and 30-day readmission over a 24-month period? **Study Design:** Retrospective cohort – feasible via EMR data pull in one semester.

*Segment 2: Project Assignment & PICOT Draft (20–25 minutes)*

Facilitator Instructions

1. Distribute project assignment summary sheets if chosen, and students should get into the group assignments or make them.
2. Circulate among students. Use the prompting questions in the table below when students are stuck.
3. Encourage students who finish early to begin thinking about their study design and what data they would need. They can also begin their literature search and background reading.

**Table:**

If the student says…	Try asking…
“I don’t know what my population is.”	“Who would your study ideally enroll? Is there a specific age group, diagnosis, or setting? Think about who you’d have to exclude.”
“My project doesn’t have an intervention.”	“That’s common in observational studies. The ‘I’ can also stand for the exposure or characteristic you’re studying – what’s the main factor your project is looking at?
“I don’t have a comparison group.”	“Descriptive studies sometimes don’t need one. But could you compare to a historical average, a different patient group, or a national benchmark? If truly not applicable, leave it blank and note why.”
“I don’t know what outcomes to measure.”	“What would tell you if the intervention or exposure ‘worked’ or mattered? What would a clinician or administrator care about? Think about mortality, LOS, time to treatment, cost, readmission, or patient satisfaction.”
“My idea seems too big to study in one semester.”	“Good instinct. Let’s scope it down – can you limit the date range, the population, or the number of outcomes? A focused, well-done small study is better than an ambitious incomplete one.”

**Facilitator Notes & Common Pitfalls**

**Table:**

Common Challenge	Facilitator Response
**Students write overly broad questions**	Help them scope it: “Let’s limit the population or outcome so it’s something you can actually collect data on this semester.”
**Students want to do a prospective/interventional study**	Validate the idea; if it’s feasible for your program help them tackle it and rework the time frame. If not, then redirect. You can sometimes break it into smaller or more preparatory projects that they can build on later or are related to their subject of interest.
**Resident mentors don’t join or dominate the discussion**	Encourage residents to use guiding questions rather than giving answers: “What would you add or change about your student’s P? What data do you have available for the I and O?”

**Table:**

Post-Workshop Checklist for Facilitators
□ Collect completed PICOT worksheets from all students (Admin RA can track). □ Note any students who did not complete or struggled significantly – follow up before Day 2/3 IRB workshop. □ Flag any projects that may require prospective data collection – discuss feasibility with resident before IRB workshop.

### Appendix L2: IRB Drafting Workshop Facilitator’s Guide

**Table:**

**Duration**	60–90 minutes
**Timing**	Day 2/3 of the PRIMER course. Follows the IRB Components Deep Dive lecture. IRB draft should be submitted by end of Week 1.
**Faculty Needed**	Lead Faculty + 1–2 Support Faculty floating between groups
**Room Setup**	Small groups
**Materials**	Your institution’s IRB protocol template (paper or digital) – one per student Completed PICOT worksheets from Day 1 workshop Project overview / background notes from resident mentor Facilitator’s IRB Annotation Key (see this guide) Internet access and login credentials for institutional IRB portal (if submitting electronically) Optional: guest speaker from institutional IRB office or university research librarian

**Learning Objectives**

By the end of this workshop, students will be able to:

- Identify the major components of an IRB protocol and explain the purpose of each.
- Apply the ethical principles of research (beneficence, justice, non-maleficence, autonomy) to justify their study’s design and safeguards.
- Classify their study into the appropriate IRB review level (exempt, expedited, or full board).
- Draft core sections of an IRB protocol for their assigned project, including background/significance, research design, and risk/benefit assessment.

**Background for Facilitators**

*IRB Basics: What Undergraduates Need to Know*

Most PRIMER research assistants will be working on studies that fall into the exempt or expedited IRB review category, particularly retrospective chart reviews. Frame the IRB for them as a protection system, not a bureaucratic hurdle. Focus on their experience even if potentially exempt in completing the process.

*IRB Review Levels*

**Table:**

Review Level	When It Applies	PRIMER Application
**Exempt**	Minimal risk; retrospective review of existing de-identified records; surveys with no identifying info; educational research.	Most retrospective chart reviews and quality improvement studies in PRIMER will qualify for exempt status.
**Expedited**	Minor risk; some identifiable data; prospective collection of biological specimens; minor procedures.	May apply if records include identifiers, or if any prospective data collection is involved.
**Full Board**	Greater than minimal risk; vulnerable populations; interventional studies with therapeutic procedures.	Unlikely for PRIMER projects; if this comes up, consult faculty and plan for a multi-semester timeline.

*Key IRB Protocol Sections*

The specific form varies by institution, but most IRB protocols include the following core sections. This annotation key is intended to help facilitators guide students as they draft.

**Table:**

Section	What It Should Include	Facilitator Prompt / Common Mistakes
**Title & Personnel**	Full study title, PI, co-investigators, contact info, department.	Remind students: the faculty member is almost always the PI. Pre-professional students are typically co-investigators or research assistants. This is not related to author order on poster presentation.
**Background & Significance**	Why is this study needed? 2–3 paragraphs. What is already known? What gap does this study fill? Cite 3–5 key references.	Students often write too much summary and too little gap identification. Ask: “Why hasn’t someone answered this question yet?”
**Research Question / Objectives**	The PICOT question in formal language. Primary and secondary objectives.	Students can copy directly from their PICOT worksheet. Walk them through converting their question into a formal objective (“The primary aim of this study is to...”)
**Study Design & Methods**	Study type, population, inclusion/exclusion criteria, data source, data collection procedure, timeline.	EMR data pulls: note that the HIT coordinator will pull data; students should describe the variables to be abstracted, not the EMR query itself.
**Subject Selection & Recruitment**	How subjects are identified. Inclusion and exclusion criteria. Estimated sample size.	For retrospective studies, this is a data pull, not recruitment. Students should state: “Subjects will be identified through retrospective EMR query…”
**Risk / Benefit Assessment**	Description of any risks to subjects. Justification that risks are minimal relative to benefits. Plans to minimize risk.	Retrospective studies using de-identified data: standard response is “minimal risk – de-identified data.” Students often leave this section blank – they still need to address it.
**Informed Consent / Waiver**	Will consent be obtained? If not, a waiver of consent must be requested and justified under 45 CFR 46.116.	Most PRIMER retrospective studies will request a waiver. Guide students to cite the four criteria: minimal risk, no adverse effects, could not practicably be done without waiver, subjects will be informed if applicable.
**Data Privacy & Security**	How data will be stored, who will have access, how it will be de-identified, data retention and destruction plan.	Connect to the Data Storage and Management lecture. Students should describe folder structure, password protection, and that only study personnel have access. Students without institutional approval will not have access to Protected Health Information (PHI) and resident/faculty will be the ones with the key if present for de-identified data.
**CITI Certification**	Documentation that all personnel have completed required CITI modules.	Verify all students completed CITI before the intensive. Have students confirm certificate upload process for your institution.

*Guided IRB Drafting Session (60 minutes)*

Setup (before students arrive at tables)

- Each project group should have: their completed PICOT worksheet, project background notes from the resident, a copy of the IRB form (print or open on laptop), and access to the IRB portal if submitting electronically.
- Faculty can be assigned if enough or floating to troubleshoot.
- Resident mentors should be seated with their student group for the full 60 minutes if possible.

Drafting Priorities by Time Block

**Table:**

Time	Focus Section	Facilitator Check-In Prompt
0–15 min	**Title, Personnel, Research Question / PICOT**: Students copy directly from their PICOT worksheet. Identify the PI (resident) and co-investigators.	“Is the PI listed correctly? Is your PICOT question in the form of a formal objective?”
15–30 min	**Background & Significance**: 2–3 paragraphs. What is known? What gap exists? Students should reference 3–5 papers from their literature search or from the resident.	“What would a clinician reading this need to know to understand why the study is worth doing?”
30–45 min	**Study Design, Population, Data Source**: Describe the study type. Identify inclusion/exclusion criteria. Name the data source and how the pull will occur.	“Is it clear who is in and who is out of your study? Who will perform the data pull?”
45–55 min	**Risk/Benefit & Consent Waiver**: Address de-identification. Draft consent waiver language or confirm exempt status applies.	“Is your data de-identified? Do you know the four criteria for a waiver?”
55–60 min	**Data Security Plan**: Where will data be stored? Who has access? How will it be protected? Connect to Data Management lecture.	“Have you mapped out your folder structure? Is the drive password-protected?”

*Facilitator Float Strategy*

With 4–8 students across 2–4 project groups, faculty should circulate rather than anchor to one group.

- Spend 10–12 minutes with each group per rotation.
- Prioritize groups without a resident mentor or groups whose projects involve identifiable data.

**Facilitator Notes & Common Pitfalls**

**Table:**

Common Challenge	Facilitator Response
**Students treat the IRB as paperwork to fill out, not a scientific document**	“The IRB isn’t a form – it’s your study design written out for someone who’s never heard of your project. If a reviewer can’t understand what you’re doing from this document alone, we need to revise it.”
**Students leave risk/benefit or consent sections blank**	“Every section has to be completed. Even if your answer is ‘minimal risk’ you still have to write it out and justify it. The IRB reviewers need to see that you thought about it.”
**The resident is not present for the drafting session**	Have students draft with available notes and flag sections requiring resident input. Follow up with the resident before the Week 1 check-in. Encourage an early meeting with the resident.
**Students don’t know the data source or feasibility of the pull**	“Write a placeholder and flag it. After today, contact your resident and the HIT coordinator to confirm what data is available. Do this in Week 1 – this is the most common delay in project completion.”
**Students confuse HIPAA de-identification with IRB exemption**	“HIPAA de-identification is about protecting patient information; IRB exemption is about the level of review your study needs. They’re related but not the same. Deidentified data often supports an exemption claim, but you still have to file and get the determination in writing.”

**Table:**

Post-Workshop Checklist for Facilitators
□ Confirm all CITI certifications are on file □ Flag any groups who did not have a resident present – ensure early follow-up meeting is scheduled □ Note any projects that may require expedited or full board review and plan accordingly □ Review submitted IRB drafts before student submits to IRB – do not let students submit without faculty review

## References

[1] Ahmed Y, Taha MH, Khayal S (2024). Integrating research and teaching in medical education: Challenges, strategies, and implications for healthcare. J Adv Med Educ Prof.

[2] Gehrke P, Binnie A, Chan SP (2019). Fostering community hospital research. CMAJ.

[3] DiDiodato G, DiDiodato JA, McKee AS (2017). The research activities of Ontario’s large community acute care hospitals: A scoping review. BMC Health Serv Res.

[4] (2023). The premed competencies for entering medical students [Internet].

[5] Koenig TW, Parrish SK, Terregino CA, Williams JP, Dunleavy DM, Volsch JM (2013). Core personal competencies important to entering students’ success in medical school: What are they and how could they be assessed early in the admission process?. Acad Med.

[6] Cline D, Henneman P, Van Ligten P (1992). A model research curriculum for emergency medicine. Ann Emerg Med.

[7] Fraker LD, Orsay EM, Sloan EP, Bunney EB, Holden JA, Hart RG (1996). A novel curriculum for teaching research methodology. J Emerg Med.

[8] Abu-Laban RB, Jarvis-Selinger S, Newton L, Chung B (2013). Implementation and evaluation of a novel research education rotation for Royal College of Physicians and Surgeons emergency medicine residents. CJEM.

[9] Oliver JJ, Ross JM, Davis WT (2019). The development of an emergency medicine resident research program in the United States Military. Mil Med.

[10] Yarris LM, Coates WC, Lin M (2012). A suggested core content for education scholarship fellowships in emergency medicine. Acad Emerg Med.

